# Antiviral Activity of Chicken Cathelicidin B1 Against Influenza A Virus

**DOI:** 10.3389/fmicb.2020.00426

**Published:** 2020-03-19

**Authors:** Lianci Peng, Wenjuan Du, Melanie D. Balhuizen, Henk P. Haagsman, Cornelis A. M. de Haan, Edwin J. A. Veldhuizen

**Affiliations:** ^1^Department of Infectious Diseases & Immunology, Division of Molecular Host Defense, Faculty of Veterinary Medicine, Utrecht University, Utrecht, Netherlands; ^2^Department of Infectious Diseases & Immunology, Division Virology, Faculty of Veterinary Medicine, Utrecht University, Utrecht, Netherlands

**Keywords:** host defense peptides, cathelicidins, influenza, innate immunity, infection

## Abstract

Cathelicidins (CATHs) are host defense peptides (HDPs) that play an important role in the innate immune response against infections. Although multiple functions of cathelicidins have been described, including direct antimicrobial activity and several immunomodulatory effects on the host, relatively little is known about their antiviral activity. Therefore, *in vitro* antiviral activity of chicken cathelicidins and the underlying mechanism was investigated in this study against different influenza A virus (IAV) strains. Our results show that chicken CATH-B1 has broad anti-IAV activity compared to other cathelicidins (CATH-1, -2, -3, LL-37, PMAP-23, and K9CATH) with an inhibition of viral infection up to 80% against three tested IAV strains (H1N1, H3N1, and H5N1). In agreement herewith, CATH-B1 affected virus-induced inflammatory cytokines expression (IFN-β, IL-1β, IL-6, and IL-8). Incubation of cells with CATH-B1 prior to or after their inoculation with virus did not reduce viral infection indicating that direct interaction of virus with the peptide was required for CATH-B1’s antiviral activity. Experiments using combined size exclusion and affinity-based separation of virus and peptide also indicated that CATH-B1 bound to viral particles. In addition, using electron microscopy, no morphological change of the virus itself was seen upon incubation with CATH-B1 but large aggregates of CATH-B1 and viral particles were observed, indicating that aggregation might be the mechanism of action reducing IAV infectivity. Neuraminidase (NA) activity assays using monovalent or multivalent substrates, indicated that CATH-B1 did not affect NA activity *per se*, but negatively affected the ability of virus particles to interact with multivalent receptors, presumably by interfering with hemagglutinin activity. In conclusion, our results show CATH-B1 has good antiviral activity against IAV by binding to the viral particle and thereby blocking viral entry.

## Introduction

Cathelicidins are short cationic peptides with an important role in the innate immune response against infections. They are mainly expressed by leukocytes and epithelial cells at infection sites in the host. Cathelicidins have been found in all vertebrates, including pig, dog, human and chicken, but with some diversity in number and structure. For example, only one 37 amino acid-long cathelicidin (LL-37) is present in human, while chicken has four cathelicidins (CATH-1, -2, -3 and -B1) with varying length. Cathelicidins have direct antimicrobial activity against a broad range of bacteria and also possess many immunomodulatory functions on host cells (Cuperus et al., 2013; van Harten et al., 2018). Out of the chicken cathelicidins, CATH-2 has been studied most. Besides having broad antibacterial activity, it can inhibit LPS-induced TLR4 activation and enhance DNA-induced TLR21 activation in macrophages (Coorens et al., 2015; Coorens et al., 2017). Furthermore, CATH-2 treatment *in ovo* has been described to reduce mortality induced by avian pathogenic *E. coli* in chicken (Cuperus et al., 2016). Less information is known about the other chicken cathelicidins. CATH-1 and CATH-3 seem to share at least the antimicrobial potency and their localization with CATH-2 (Veldhuizen et al., 2013; Sekelova et al., 2017). On the contrary, the function of CATH-B1 is hardly studied, but it is different from CATH1-3 by its localization in the bursa of Fabricius in chicken (Goitsuka et al., 2007). In addition, the antiviral activity for all chicken cathelicidins is still unknown.

Influenza A virus (IAV) is an important pathogen of human and animals. Infection with IAV causes acute respiratory diseases leading to morbidity and mortality in human and many animal species. In the past 100 years, influenza A viruses, such as H1N1 in 1918 and H3N2 in 1968, have caused severe pandemics in human (Cox and Subbarao, 2000; Webby and Webster, 2003). Animal IAVs, such as highly pathogenic IAV H5N1, pose a constant threat of causing a new pandemic. This latter virus has been reported to infect humans with a mortality rate of 52.8% from 2003-2019 (source: WHO). Moreover, due to rapid genomic variation of IAVs, novel variants are emerging (such as H7N9 in 2013) that pose a new threat to human health (Khazeni et al., 2014). Currently, vaccination and anti-IAV drugs are being used to prevent and treat IAV infections. The efficacy of vaccination is, however, limited in part due to antigenic variation, while the use of anti-IAV drugs is limited by the development of resistance. Therefore, novel preventive and therapeutic options against IAV infection are needed.

In this study, we investigated the antiviral activity and mechanism of chicken cathelicidins against IAVs. The outcome of our study provides useful information for the development of therapies against IAV infection.

## Materials and Methods

### Peptides

All the peptides were synthesized by China Peptides (Shanghai, China) using Fmoc-chemistry. All peptides were purified by reverse phase high-performance liquid chromatography to a purity >95%.

### Cell Lines and Viruses

HD11 cells (a chicken macrophage cell line) and Madin–Darby Canine kidney (MDCK-II; ATCC) cells were cultured in RPMI 1640-glutaMAX and DMEM-glutaMAX (Gibco, United Kingdom), respectively, supplemented with 10% FCS and antibiotics (100 U/ml penicillin and 100 μg/ml streptomycin).

Influenza virus A/Puerto Rico/8/34/Mount Sinai (H1N1/PR8) and reassortant viruses were propagated in MDCK-II cells as described previously and stored aliquoted at −80°C until use. Generation of reassortant viruses H3N1 (containing the HA gene from A/Bilthoven/1761/76 (H3N2) in the genetic background of PR8) and H5N1 (containing the HA gene from A/duck/Hunan/795/2002 (H5N1) in the genetic background of PR8) was described previously (Koel et al., 2013; Peeters et al., 2017). The H3N1 virus was kindly provided by Ron Fouchier (Erasmus Medical Center, Netherlands). Virus titers were determined for MDCK-II cells by calculating 50% tissue culture infectious dose per ml (TCID50/mL) as described before (Reed and Muench, 1938).

### Viral Infection

MDCK-II and HD11 cells, seeded in 96 well plates and grown to confluency, were infected with virus at a multiplicity of infection (MOI) of 0.1 in the presence or absence of cathelicidins for 1 h at 37°C. In pre-incubation studies CATHs were added to the cells for 1 h, washed away with PBS after which IAV was added for 1 h. Post incubation studies were performed similarly but with the order of peptide and virus addition reversed. All the initial infection and cathelicidin incubation steps were performed in the absence of serum. After these incubations, unbound virus or unbound peptide were removed by washing the cells twice with PBS (supplemented with Ca^2+^ and Mg^2+^). MDCK-II and HD11 cells were incubated for another 7 h with opti-MEM or RPMI 1640-glutaMAX supplemented with 2% FCS, respectively, at 37°C. Subsequently, cells were fixed with cold methanol at −20°C for 5 min, after which cells were stained with primary mouse monoclonal antibody HB65 (1:1000) specific for the nucleoprotein and Alexa Fluor 488-labeled Donkey anti-Mouse IgG antibodies (Life technologies, Eugene, OR, United States) (1:1000) as described previously (De Vries et al., 2011). Cells were visualized using the nuclear stain DAPI (Thermo Fischer Scientific) according to the manufacturer’s instructions. Three images per well were taken using an EVOS FL microscope (Thermo Fisher Scientific) and the infected cells were counted. The number of infected cells in inoculated, mock-treated wells was set at 100%.

To investigate whether a direct interaction of CATH-B1 with virus was present, and possibly required for CATH-B1’s activity, Capto Core 700 beads (GE Healthcare) were used to remove CATH-B1 that was not bound to virus. To this end, viruses were pre-incubated in opti-MEM medium with or without CATH-B1 for 30 min at 37°C, after which Capto Core 700 beads were added to the samples and samples were incubated for 20 min at 4°C while rotating. Afterward, beads were spun down and supernatants were collected. To control for the efficient removal of CATH-B1, samples containing CATH-B1 but no virus were subjected to the same procedure. Cells were inoculated with the supernatants (or combinations thereof) and processed to determine the number of infected cells as described above.

### Cell Viability

Cell viability was determined using the WST-1 assay following the manufacturer’s protocol. In short, cells were incubated with peptides for 1 h at 37°C, then peptides were washed away and cells were further incubated for either 7 h or 23 h at 37°C (corresponding to the incubation times used for immunohistochemistry and detection of cytokine gene expression, respectively). Cell culture medium was removed and replaced with fresh culture medium containing 10% WST-1 reagent. After 20 min incubation, absorbance was measured at 450 nm with a FLUOstar Omega microplate reader and was corrected for absorbance at 630 nm.

### Electron Microscopy

Influenza A virus (H3N1) was incubated in the presence or absence of CATH-B1 for 1 h at 37°C and 10 μl sample was placed on a carbon-coated copper grid. Grids were washed three times with PBS and fixed with 1% glutaraldehyde (Sigma-Aldrich) in PBS for 10 min. Next, grids were washed two times with PBS and four times with MilliQ. Subsequently, grids were shortly rinsed with methylcellulose/uranyl acetate (pH 4) and incubated for 5 min with methylcellulose/uranyl acetate (pH 4) on ice. Finally, grids were looped out of the solution and air-dried. Samples were imaged on a Tecnai-12 electron microscope (FEI).

### MUNANA and ELLA Assay

The activity of NA in the presence of CATH-B1 toward the synthetic monovalent substrate 2′-(4-methylumbelliferyl)-alpha-D-N-acetylneuraminic acid (MUNANA) (Sigma-Aldrich) was determined using a fluorometric assay similarly to what was described previously (Dai et al., 2017). In short, IAV was incubated with CATH-B1 (0–40 μM) for 1 h at 37°C, followed by addition of MUNANA for another 1 h at 37°C. Next, the reaction was stopped, and fluorescence intensity was measured using a FLUOstar Omega microplate reader. The activity of NA toward the sialylated glycoprotein fetuin was analyzed in a solid phase cleavage assay using a previously described enzyme linked lectin assay (ELLA) (Dai et al., 2017; Du et al., 2019). Fetuin (2.5 ug/mL) was coated on Maxisorp Nunc 96-well plates (Thermo Fisher Scientific). Plates were incubated with IAV PR8 (1.78 × 10^8^ PFU/mL) in the presence or absence of 5 μM CATH-B1 (in 50 mM Tris–HCl with 4 mM CaCl_2_, pH = 6) for 2 h at 37°C. Subsequently, the plates were washed three times with PBS/0.05% Tween 20 after which terminal galactose moieties were quantified using biotin-conjugated peanut agglutinin E. Cristagalli (ECA) lectin (Vector laboratories) (1.5 μg/ml) in combination with streptavidin-HRP (Thermo Fisher Scientific) (1:1000). After washing, TMB was added and plates were incubated for 1–4 min at room temperature. Sulfide acid (25%) was used to stop the reaction. Finally, the plate was read at OD450 nm using the FLUOstar Omega microplate reader. Final OD450 nm values are presented as OD450 nm_*sample*_-OD450 nm_*backgroud*_.

### Quantitative Real-Time PCR (qPCR)

HD11 cells were infected with virus for 1 h at the multiplicity of infection (MOI) of 1 in the presence or absence of CATH-B1 as described above. After 8 or 24 h incubation, total RNA was extracted by Trizol (Ambion, Carlsbad, CA, United States) reagent according to the manufacturer’s instructions. RNA (500 ng) was reverse transcribed by the iScript cDNA synthesis kit (Bio-Rad, Veenendaal, Netherlands) according to the manufacturer’s instructions. Primers and probes were designed and produced by Eurogentec (Seraing, Belgium) (Table 1). Quantitative real time PCR was performed on a CFX Connect qPCR with CFX Manager 3.0 (Bio-Rad). Reactions were performed as follows: 3 min at 95°C; 40 cycles: 10 s at 95°C, 30 s at 60°C and 30 s at 72°C. Relative gene expression levels were normalized against the expression levels of the house keeping gene GAPDH.

**TABLE 1:** Primer and probe sequences for qPCR.

**Gene**		**5′→3′sequence**
GAPDH	Forward	GTCAACCATGTAGTTCAGATCGATGA
	Reverse	GCCGTCCTCTCTGGCAAAG
	Probe	AGTGGTGGCCATCAATGATCCC
IFN-a	Forward	GACAGCCAACGCCAAAGC
	Reverse	GTCGCTGCTGTCCAAGCATT
	Probe	TCCACCGCTACACCCAGCAGCACCTCG
IFN-β	Forward	CCTCCAACACCTCTTCAACACG
	Reverse	TGGCGTGTGCGGTCAAT
	Probe	AGCAGCCCACACACTCCAAAACACT
IL-1β	Forward	GCTCTACTAGTCGTGTGTGATGAG
	Reverse	TGTCGATGTCCCGCATGA
	Probe	CCACACTGCAGCTGGAGGAAGCC
IL-6	Forward	GTCGAGTCTCTGTGCTAC
	Reverse	GTCTGGGATGACCACTTC
	Probe	ACGATCCGGCAGATGGTGA
IL-8	Forward	GCCCTCCTCCTGGTTTCA
	Reverse	CGCAGCTCATTCCCCATCT
	Probe	TGCTCTGTCGCAAGGTAGGACGCTG

### Statistical Analysis

Data are represented as mean ± SEM of three independent experiments for each group (*n* = 3) and were analyzed by a *T*-test for two groups or by one-way ANOVA with Tukey’s multiple comparisons test for more than two groups. Bio-Rad CFX Manager 3.0 software was used for qPCR data analysis. All graphs were made using GraphPad Prism 5.0.

## Results

### Cytotoxicity and Anti-IAVs Activity of Cathelicidins

**FIGURE 1:**
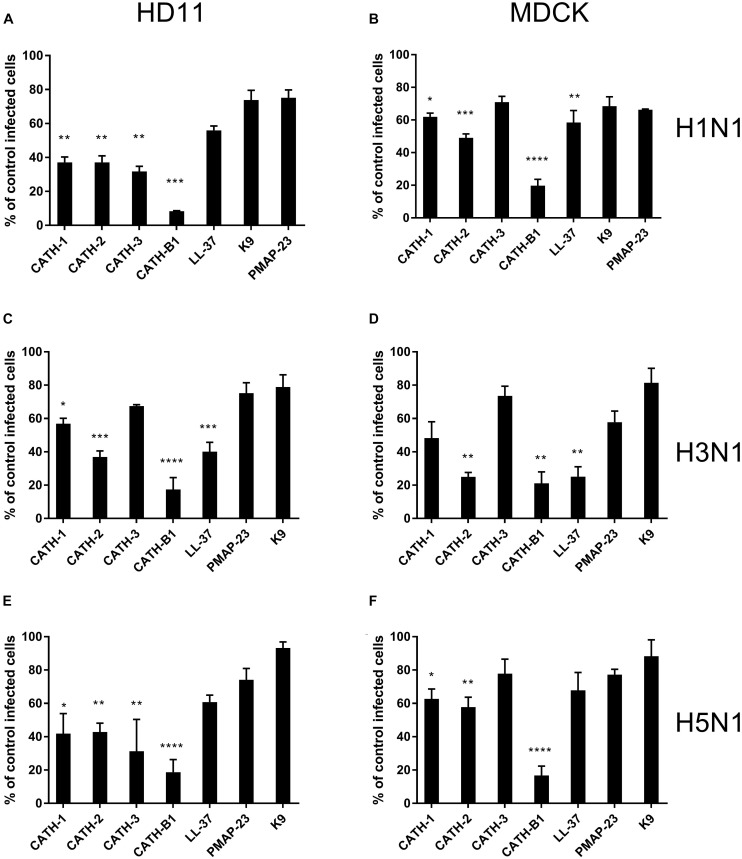
The antiviral effect of cathelicidins against 3 IAV strains (H1N1/PR8, H3N1 and H5N1). Cathelicidins were mixed with virus strains before addition to either HD11 or MDCK cells. H1N1/PR8 infection in the presence of cathelicidins of HD11 **(A)** or MDCK **(B)** cells. H3N1 infection in the presence of cathelicidins of HD11 **(C)** or MDCK **(D)** cells. H5N1 infection in the presence of cathelicidins of HD11 **(E)** and MDCK **(F)** cells. Viral infection was determined by immunofluorescent detection of IAV nuclear protein. Three images per well were taken and the infected cells were counted. The infection rate in the presence of cathelicidins was normalized against only virus-treated wells. Data are represented as mean ± SEM of three independent experiments of triplicate samples per experiment. *p ≤ 0.05;**p ≤ 0.01;***p ≤ 0.005; ****p ≤ 0.001.

To investigate the anti-IAV activity of cathelicidins, three IAV strains (H1N1/PR8, H3N1 and H5N1) were used in this study. Both HD11 and MDCK cells were inoculated with IAVs in the presence or absence of 5 μM cathelicidins for 1 h. After 1 h, viruses and peptides were removed, and cells were incubated for another 7 h. At 8 hours post infection (hpi), the number of infected cells was quantified by immunofluorescent labeling of the influenza nuclear protein. As shown in Figure 1, the cathelicidins displayed different antiviral activities, which for some of them depended to some extent on the viral strain and the cell line used. PMAP-23 and K9 did not significantly inhibit infection. Interestingly, LL-37 only showed activity against H3N1 and to a lower extent H1N1 but not against H5N1, while the chicken cathelicidins were active against all three influenza strains with inhibition of infectivity of 40–70%. However, regardless of the cell line or viral strain used, CATH-B1 clearly displayed the strongest antiviral activity, inhibiting infection up to 80–90%.

**FIGURE 2:**
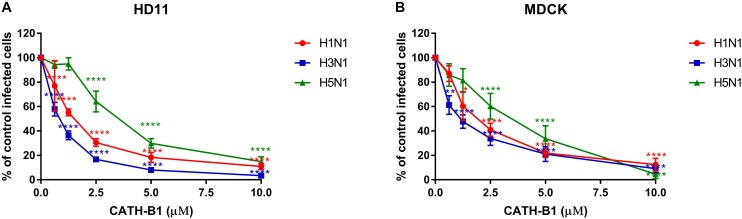
Dose-dependent antiviral activity of CATH-B1 against IAV strains (H1N1/PR8, H3N1 and H5N1). **(A)** Viral infection in the presence of CATH-B1 in HD11 cells, **(B)** Viral infection in the presence of CATH-B1 in MDCK cells. Viral infection was determined by immunofluorescent detection of IAV nuclear protein. Three images per well were taken and the infected cells were counted. The infection rate in the presence of cathelicidins was normalized against the virus only control wells. Data are represented as mean ± SEM of two independent experiments of triplicate samples per experiment. *p ≤ 0.05;**p ≤ 0.01;***p ≤ 0.005; ****p ≤ 0.001.

**FIGURE 3:**
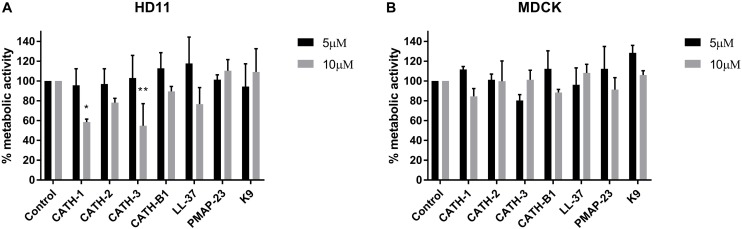
Cytotoxicity of cathelicidins. HD11 and MDCK cells were incubated with cathelicidins, and metabolic activity was tested using WST-reagent. **(A)** Metabolic activity of HD11 cells incubated for 24 h with cathelicidins. **(B)** Metabolic activity of MDCK cells incubated for 8 h with cathelicidins. Data are represented as mean ± SEM of three independent experiments of triplicate samples per experiment. *p ≤ 0.05; **p ≤ 0.01.

The inhibitory infectivity of CATH-B1 was dose-dependent (Figure 2), with an almost complete inhibition of viral infectivity of the H1N1 and H3N1 strains, while inhibition of H5N1 reached 85% (Figure 2). The observed reduction in infected cells was not due to toxicity of cathelicidins toward the mammalian cell lines as shown by the WST assay (Figure 3). Nevertheless, cytotoxicity was observed for CATH-1 and CATH-3 at 10 μM to HD11 cells (Figure 3A).

When cathelicidins and virus were sequentially added to cells (either pre- or post-incubation of peptide relative to virus inoculation), the inhibitory effect was mostly lost (Supplementary Figure S1). This indicates that the antiviral effect of the peptides was not achieved through interaction with the HD11 or MDCK cells or by an inhibitory effect on viral replication after the viruses entered the cells, but that CATH-B1 likely blocked viral entry to the cells by direct interaction with the virus.

### The Effect of CATH-B1 on IAV-Induced Gene Expression of Cytokines in HD11 Cells

Activation of macrophages is important for viral clearance during IAV infection, but an excessive inflammatory response might cause morbidity and mortality (Cheung et al., 2002; Kim et al., 2008; Högner et al., 2013). As several cathelicidins have been reported to affect innate immune responses (Coorens et al., 2015; Coorens et al., 2017), we analyzed to what extent the presence of CATH-B1 affected these responses induced by infection of cells with IAV. To this end, virus-induced gene expression of cytokines in HD11 macrophages was determined by qPCR in the presence or absence of CATH-B1.

**FIGURE 4:**
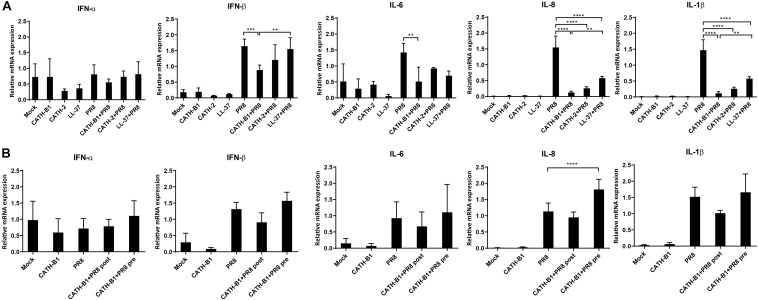
The effect of CATH-B1 on PR8-induced immune response in HD11 cells. **(A)** Cytokine expression in HD11 cells at 24 hpi in the presence or absence of peptides. **(B)** Cytokine expression in HD11 cells for pre- or post-incubation with CATH-B1. Relative gene expression levels were normalized against the expression levels of the house keeping gene GAPDH. Data are represented as mean ± SEM of two or three independent experiments of triplicate samples per experiment. *P ≤ 0.05;**P ≤ 0.01;***P ≤ 0.005; ****P ≤ 0.001.

Virus infection resulted in induced gene expression of IFN-β, IL-1β, IL-6, and IL-8, but surprisingly not IFN-α. However, whether this lack of IFN-α gene expression was IAV strain specific was not further investigated. CATH-B1 downregulated PR8-induced gene expression of IFN-β, IL-1β, IL-6, and IL-8, but the relative mRNA level of IFN-α was unaffected (Figure 4A and Supplementary Figure S2). CATH-2 and LL-37 also showed similar effects on gene expression upon virus infection, but the inhibition was not as pronounced as observed for CATH-B1, correlating with the effect of the peptides on virus infection shown in Figure 1. The effect of CATH-B1 on virus-induced gene expression was diminished when the cells were incubated with CATH-B1 prior to, or immediately after virus infection (Figure 4B), indicating that the reduction of the response results from the ability of the peptide to inhibit infection.

### The Interaction of CATH-B1 With IAV

**FIGURE 5:**
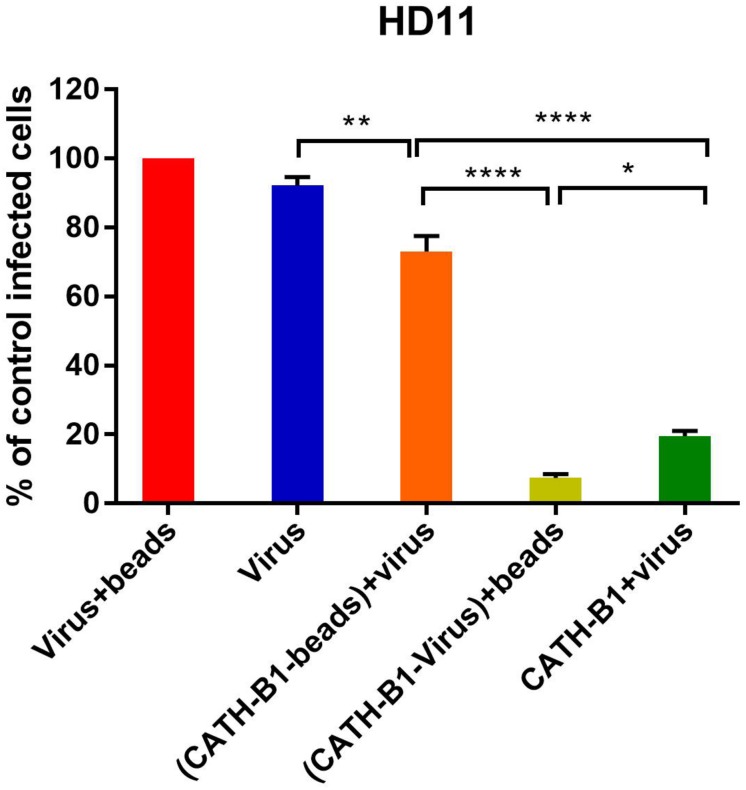
Binding of CATH-B1 to PR8 virus. CATH-B1 was pre-incubated with H1N1 virus after which peptide and virus were separated using Capto beads. (Virus containing) supernatant was then used to infect HD11 cells. Viral infection was determined by immunofluorescent detection of IAV nuclear protein. Three images per well were taken and the infected cells were counted. The infection rate in the presence of CATH-B1 was normalized against virus-only control wells. Data are represented as mean ± SEM of three independent experiments of triplicate samples per experiment. *p ≤ 0.05;**p ≤ 0.01;***p ≤ 0.005; ****p ≤ 0.001.

The inhibitory effect of CATH-B1 on virus infection and induction of cytokine responses is only observed when the peptide is present during inoculation of cells with virus, but not when cells are exposed to the peptides prior to, or immediately after virus infection. This suggests that a direct interaction of the peptide with the virus is required for its antiviral effect. To further investigate the antiviral mechanism of CATH-B1, a series of experiments was performed using H1N1. Firstly, a crucial role for a direct interaction of CATH-B1 with virus particles was analyzed by removal of unbound CATH-B1 using Capto Core 700 beads. As controls, incubation of virus itself with the beads did not affect virus infectivity (Figure 5, red bar), while addition of CATH-B1 solution to virus preparations again resulted in 80% reduction of virus infectivity on HD11 cells (Figure 5, dark green bar). Incubation of CATH-B1 with beads prior to virus addition resulted in very little antiviral effect (Figure 5, orange bar), indicating that CATH-B1 was efficiently removed from solution by the beads. However, when CATH-B1 and virus were mixed prior to their treatment with Capto Core 700 beads, the antiviral activity of CATH-B1 was maintained indicating that CATH-B1 is directly associated with the virus and not captured by the beads (light green bar, Figure 5). The proposed binding of CATH-B1 to virus is almost instantaneous because in the absence of the 30 min incubation time upon mixing of virus and CATH-B1 prior to addition of the beads, a similar antiviral activity of CATH-B1 was observed (data not shown). Similar results were obtained using MDCK cells (Supplementary Figure S3).

### The Effect of CATH-B1 on Morphology of Virus

**FIGURE 6:**
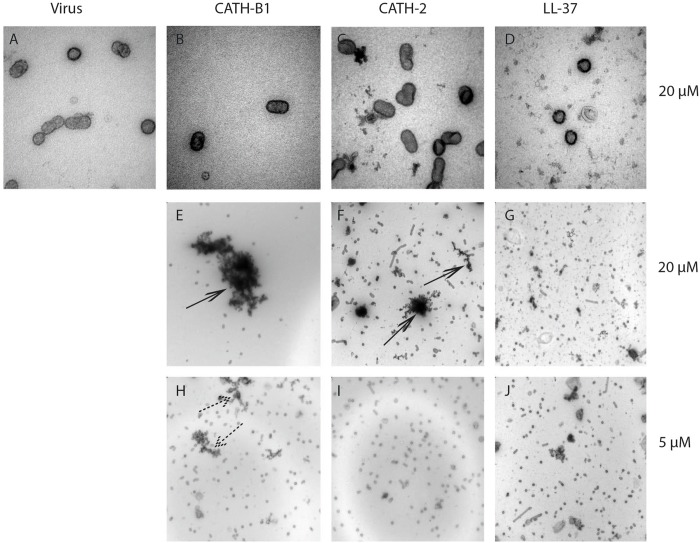
The effect of CATH-B1 on viral morphology. Representative electron microscopic images of **(A)** H3N1 IAV alone **(B–G)** IAV pretreated with 20 μM and **(H–J)** 5 μM of peptides. Shown are representative images at 60,000x magnification **(A–D)** to visualize morphology of individual virus particles, or 16,500 × magnification **(E–J)** to visualize aggregation of viral particles. Large peptides aggregates (black arrows) containing viruses and small aggregates (dashed arrows) were visible at high concentration and low concentration of peptides, respectively.

Some host defense peptides, such as human neutrophil defensins, have been shown to induce viral aggregation, which might contribute to their antiviral activity (Hartshorn et al., 2006; Doss et al., 2009). Other peptides such as LL-37 have been found to directly disrupt the viral membrane (Tripathi et al., 2013). To study the effect of CATH-B1 on viral morphology, H3N1 was used in this study as an example. As shown in Figure 6, there is no clear alteration of the viral structure upon incubation with 20 μM CATH-B1 (Figure 6B), CATH-2 (Figure 6C), or LL-37 (Figure 6D), However, large aggregates were observed that contained viral particles and electron dense material at high concentration (20 μM) of CATH-B1 (Figure 6E), while some smaller aggregates were observed at this concentration for CATH-2 and LL-37 (Figures 6F,G). At 5 μM CATH-B1, smaller aggregates were observed (Figure 6H), but not for the two other peptides (Figures 6I,J). These results indicate that binding and aggregation of virus particles is likely involved in the antiviral mechanism of CATH-B1.

### The Effect of CATH-B1 on Hemagglutinin and Neuraminidase Activity

**FIGURE 7:**
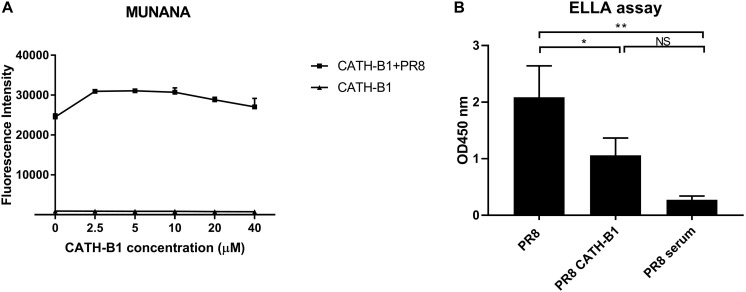
The effect of CATH-B1 on HA and NA activity. **(A)** NA activity of H1N1/PR8 was directly measured using MUNANA substrate in presence or absence of CATH-B1. **(B)** Desialylated N-glycans of fetuin were detected using HRP-conjugated ECA lectins, after incubation with H1N1/PR8 in presence or absence of CATH-B1. Data are represented as mean ± SEM of two independent experiments of triplicate samples per experiment. *p ≤ 0.05;**p ≤ 0.01.

Hemagglutinin (HA) and neuraminidase (NA) are important functional proteins on the surface of IAVs. During viral infection, the function of HA is binding to sialic acid receptors on host cells and subsequent membrane fusion, while release of newly assembled virus particles requires the sialidase activity of NA. First, we analyzed the ability of CATH-B1 to interfere with the receptor-binding properties of HA by performing a hemagglutination inhibition assay. Unfortunately, CATH-B1 to some extent induced lysis of erythrocytes, which precluded further analysis of the hemagglutination inhibition assay. Next, we analyzed the ability of CATH-B1 to interfere with NA activity using the substrate MUNANA. Clearly, even at the highest CATH-B1 concentrations, no inhibition of NA activity was observed (Figure 7A). Next we performed a solid phase cleavage (ELLA) assay using the glycoprotein fetuin. Cleavage of fetuin by NA in this assay depends on the activity of NA, but also on the activity of HA, as receptor-binding by HA contributes significantly to NA cleavage (Guo et al., 2018; Lai et al., 2019), at least when multivalent receptors are used. CATH-B1 inhibited cleavage of sialic acids on fetuin (Figure 7B). As CATH-B1 did not affect NA activity *per se* as demonstrated with the MUNANA assay, we conclude that the inhibitory effect in the ELLA assay results from the ability of CATH-B1 to interfere with virus-receptor binding.

## Discussion

Cathelicidins are important peptides of the innate immune system that protect against invading pathogens. Although cathelicidins have mostly been studied with respect to their antibacterial activity, more recently, studies show potential antiviral activity of these peptides. For example, the human cathelicidin LL-37 has been found to have antiviral activity against IAV, adenovirus, respiratory syncytial virus and HIV (Bergman et al., 2007; Barlow et al., 2011; Currie et al., 2016). In addition, defensins including α- and β-defensins have also been found to exhibit antiviral activity against IAV (Doss et al., 2009).

Chicken cathelicidins have been studied quite extensively and they have shown to possess many different activities. Besides broad spectrum antibacterial activity, they enhance phagocytosis, neutralize LPS-induced immune responses and enhance DNA-induced TLR21 activation (Veldhuizen et al., 2013; Coorens et al., 2015; Coorens et al., 2017). However, antiviral activity of chicken cathelicidins was never described. Therefore, we investigated the anti-IAV activity and mechanism of inhibition of chicken cathelicidins, since IAV is an important pathogen causing disease in chicken and also in humans.

Four chicken cathelicidins (CATH-1,-2,-3,-B1) were used in this study together with a porcine (PMAP-23), canine (K9CATH) and human cathelicidin (LL-37) for comparison. Our results showed that CATH-B1 has the strongest anti-IAV activity against all three tested virus strains in this study. Comparison of the peptides does not give a clear indication what the main determinant for antiviral activity might be. All peptides have a (predicted) helical structure, are highly cationic and amphipathic. However, the sequence homology itself is quite low between peptides (except for CATH-1 and CATH-3 that also seem to have comparable activity). CATH-B1 is slightly longer than the other cathelicidins tested but it is unclear if that contributes to antiviral activity. Only for LL-37 some structure- antiviral activity studies have been performed which indicated that the central 20 amino acid fragment of LL-37 played a critical role in inhibiting the infection IAV (Tripathi et al., 2015). Future mutational studies on CATH-B1 could indicate which domains or residues are important for its observed activity against IAV.

CATH-B1 is different from the other three chicken cathelicidins in several ways. Besides some structural and sequence differences (Table 2), it was reported to be exclusively present in the bursa of Fabricius. The peptide is expressed by secretory epithelial cells but is located after secretion surrounding bursal M-cells (Goitsuka et al., 2007). In contrast, CATH-1,-2, and -3 are mostly expressed in the bone marrow and at least for CATH-2 it was shown that it is present in specific granules in heterophils (Van Dijk et al., 2009; Sekelova et al., 2017) where the peptide is released upon infection (Van Dijk et al., 2009). In order to determine if CATH-B1 is important *in vivo* against viral infections, more detailed studies on its expression and localization are needed. If CATH-B1 is indeed only present in the bursa, only a limited antiviral role against for example infectious bursal disease virus can be envisioned, but not really against IAV or other repiratory or intestinal viruses. However, CATH-B1 gene expression seems not restricted to the bursa of Fabricius. Although at much lower levels than found in the bursa, CATH-B1 mRNA was present in several tissues, including spleen and multiple segments of the respiratory and gastrointestinal tract of chicken (Achanta et al., 2012; Rodriguez-Lecompte et al., 2016). CATH-B1 gene expression was also observed in chicken HD11 macrophages and primary monocytes (Sunkara, 2011). Finally, some studies have described induced gene-expression upon LPS and LTA stimulation *in vitro*, indicating that higher CATH-B1 levels in multiple tissues upon viral infection could be obtained.

**TABLE 2:** Characteristics of peptides used in this study.

**Peptide**	**Amino acid sequence**	**Length**	**Charge**
CATH-1	RVKRVWPLVIRTVIAGYNLYRAIKKK	26	+8
CATH-2	RFGRFLRKIRRFRPKVTITIQGSARF	26	+9
CATH-3	RVKRFWPLVPVAINTVAAGINLYKAIRRK	29	+7
CATH-B1	PIRNWWIRIWEWLNGIRK RLRQRSPFYVRGHLNVTSTPQP	40	+7
LL-37	LLGDFFRKSKEKIGKEFK RIVQRIKDFLRNLVPRTES	37	+6
PMAP-23	RIIDLLWRVRRPQKPKFVTVWVR	23	+6
K9	RLKELITTGGQKIGEKIRRIGO RIKDFFKNLQPREEKS	38	+6

Human neutrophil peptides as antiviral therapeutics are gaining interest with the increasing knowledge on their antiviral potential. However, the antiviral mechanism of action can be quite different from one HDP to another, and is also depended on viruses. Human cathelicidin LL-37 has been found to directly interact with the IAV virion thereby limiting viral replication and virus-induced inflammation *in vivo* (Barlow et al., 2011). *In vitro*, LL-37 was described to directly induce disruption of the IAV viral membrane (Tripathi et al., 2013), although we did not observe this in our current study, possibly related to differences in the viral strains used. Human neutrophil peptides (HNPs) have been shown to induce viral aggregation and inhibit infectivity mainly through direct interactions with virus without any inhibition of HA activity of IAV (Hartshorn et al., 2006). Another group of HDPs, defensins, also showed antiviral activity against IAV and HIV-1 but mainly through immunomodulatory effects during viral infection (Wang et al., 2004; Ryan et al., 2011). The current study showed that CATH-B1 binds to viral particles but this was not accompanied by any obvious disruption of the viral membrane. Instead, peptide-virus aggregates were observed using electron microscopy, indicating that CATH-B1 might exert this mechanism of aggregating pathogens to block infection for viral invasion.

The viral membrane of IAV is characterized by the two key proteins on the surface of the virus, hemagglutinin (HA) and neuraminidase (NA), both of which are important for IAV infection and could potentially be affected by binding of CATH-B1 to the viral surface. HA functions as a receptor binding and fusion protein, while the NA protein is involed in release of (nascent) virus particles from decoy receptors or the cell surface (Gamblin and Skehel, 2010; Dou et al., 2018). Recently, it has been reported that NA activity of IAV is influenced by virus-receptor binding (Guo et al., 2018; Du et al., 2019; Lai et al., 2019). NA activity is altered based on enhanced or a reduced HA-receptor binding property. Our results suggest that CATH-B1 did not affect NA activity but rather inhibited virus-receptor binding activity, in agreement with CATH-B1 only affecting virus infection when present during virus inoculation. Presumably the inhibiting effect on virus-receptor interaction is related to CATH-B1 induced aggregation of virus. Whether this phenomenom results from a direct interaction of CATH-B1 with HA remains to be established, and other or additional antiviral mechanisms, such as the interaction of CATH-B1 with the viral membrane should be explored further. This antiviral mechanism of CATH-B1 appears to differ from that of LL-37. LL-37 bound to virus but did not inhibit HA-receptor binding and failed to inhibit virus binding to and uptake into cells (Tripathi et al., 2013; Tripathi et al., 2015). Moreoever, the inhibitory activity of LL-37 depends on the IAV strain used which is consistent with our observation that LL-37 showed much more antiviral activity against H3N1 than against H1N1 and H5N1. Of note, CATH-B1 showed broad antiviral activity against IAVs carrying different HA proteins.

In conclusion, this study showed the potential of CATH-B1 to bind and inhibit the infectivity of IAV, likely by interfering with HA-mediated virus-receptor binding and thereby blocking viral entry. This new activity is important to understand the *in vivo* role of this cathelicidin, but might also have important implications for the future development of new antivirals based on cathelicidins in general.

## Data Availability Statement

All datasets generated for this study are included in the article/Supplementary Material.

## Author Contributions

LP performed the experiments and wrote the manuscript. WD assisted with the experiments, MB performed the EM studies. HH, EV, and CH contributed to the supervision and wrote/corrected the manuscript.

## Conflict of Interest

The authors declare that the research was conducted in the absence of any commercial or financial relationships that could be construed as a potential conflict of interest.

## Supplementary Material

FIGURE S1 The effect of pre-incubation or post-incubation of cathelicidins on viral replication of H1N1/PR8 strain. PR8 infection in HD11 cells **(A)** or MDCK cells **(B)** for pre-incubation with cathelicidins. PR8 infection in HD11 cells **(C)** or MDCK cells **(D)** for post-incubation with cathelicidins. Viral infection was determined by immunofluorescent detection of IAV nuclear protein. Three images per well were taken and the infected cells were counted. The infection rate in the presence of cathelicidins was normalized against virus-only control wells. Data are represented as mean ± SEM of two independent experiments of triplicate samples per experiment.

FIGURE S2 The effect of CATH-B1 on PR8-induced immune response in HD11 cells. Cytokine expression in HD11 cells at 8 hpi in the presence or absence of peptides. Relative gene expression levels were normalized against the expression levels of the house keeping gene GAPDH. Data are represented as mean ± SEM of three independent experiments of triplicate samples per experiment. ^∗^*p* ≤ 0.05; ^∗∗^*p* ≤ 0.01; ^∗∗∗^*p* ≤ 0.005; ^****^*p* ≤ 0.001.

FIGURE S3 Binding of CATH-B1 to PR8 virus. CATH-B1 was pre-incubated with H1N1 virus after which peptide and virus were separated using Capto beads. (Virus containing) supernatant was then used to infect MDCK cells. Viral infection was determined by immunofluorescent detection of IAV nuclear protein. Three images per well were taken and the infected cells were counted. The infection rate in the presence of cathelicidins was normalized against only virus-treated wells. Data are represented as mean ± SEM of three independent experiments of triplicate samples per experiment ^∗^*p* ≤ 0.05; ^∗∗^*p* ≤ 0.01; ^∗∗∗^*p* ≤ 0.005; ^****^*p* ≤ 0.001.
