# Supplementary figures and images for: Antiviral Activity of Chicken Cathelicidin B1 Against Influenza A Virus
Source: Front Microbiol. 2020 Mar 19;11:426. doi: 10.3389/fmicb.2020.00426 (PMC7096384; doi:10.3389/fmicb.2020.00426)

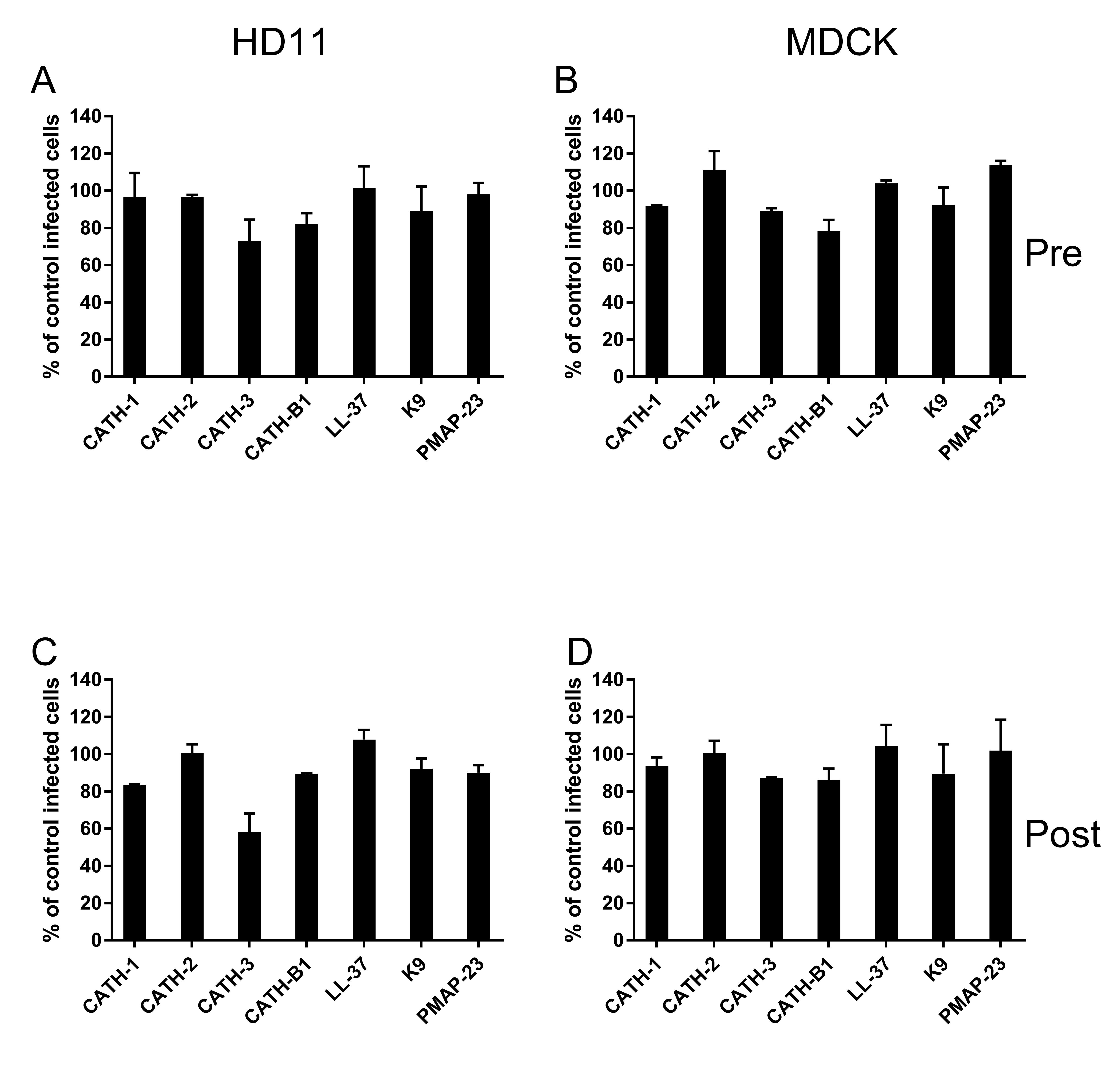

Supplement: FIGURE S1 — The effect of pre-incubation or post-incubation of cathelicidins on viral replication of H1N1/PR8 strain. PR8 infection in HD11 cells (A) or MDCK cells (B) for pre-incubation with cathelicidins. PR8 infection in HD11 cells (C) or MDCK cells (D) for post-incubation with cathelicidins. Viral infection was determined by immunofluorescent detection of IAV nuclear protein. Three images per well were taken and the infected cells were counted. The infection rate in the presence of cathelicidins was normalized against virus-only control wells. Data are represented as mean ± SEM of two independent experiments of triplicate samples per experiment. [file Image_1.JPEG]

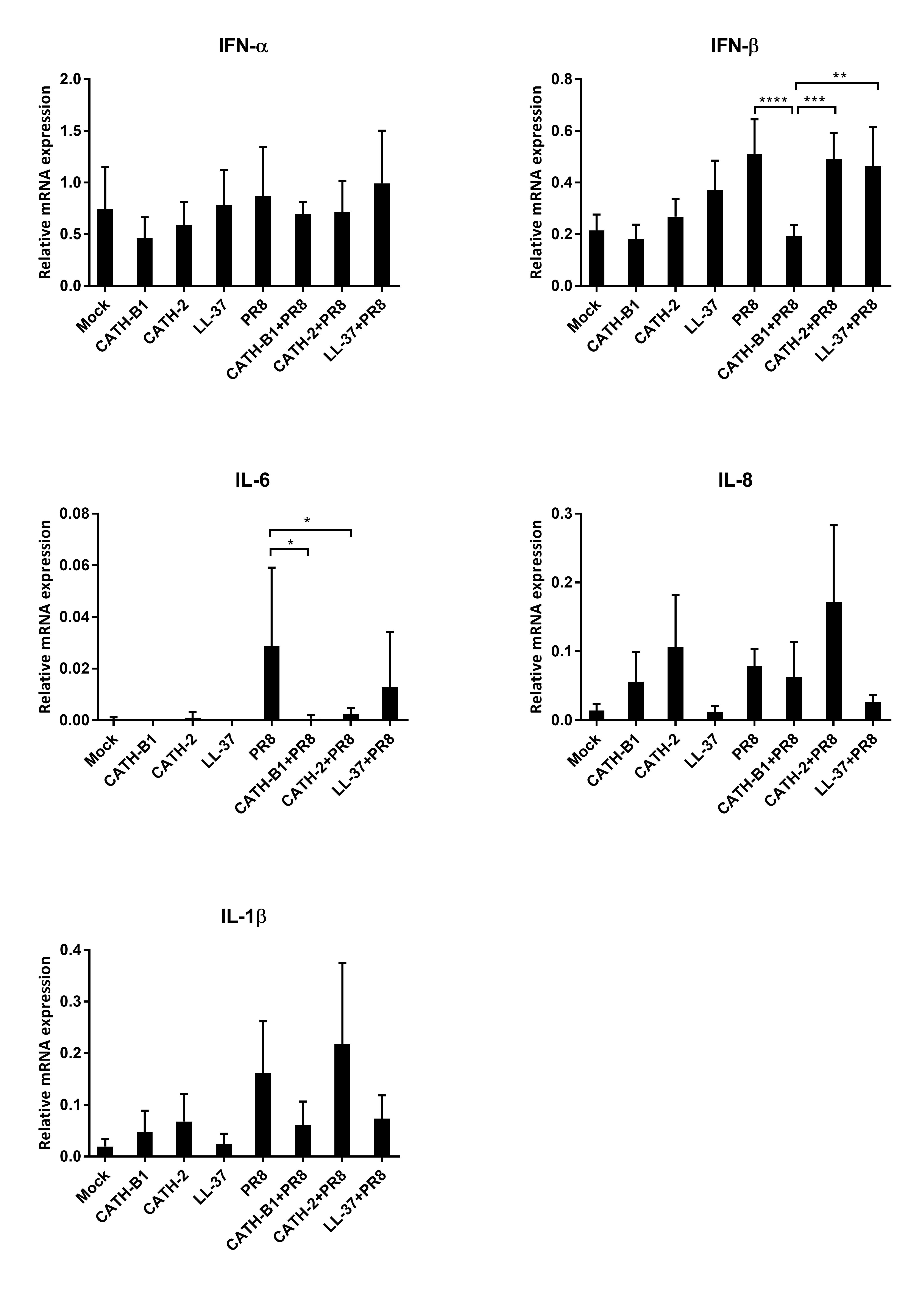

Supplement: FIGURE S2 — The effect of CATH-B1 on PR8-induced immune response in HD11 cells. Cytokine expression in HD11 cells at 8 hpi in the presence or absence of peptides. Relative gene expression levels were normalized against the expression levels of the house keeping gene GAPDH. Data are represented as mean ± SEM of three independent experiments of triplicate samples per experiment. ∗p ≤ 0.05; ∗∗p ≤ 0.01; ∗∗∗p ≤ 0.005; ****p ≤ 0.001. [file Image_2.JPEG]

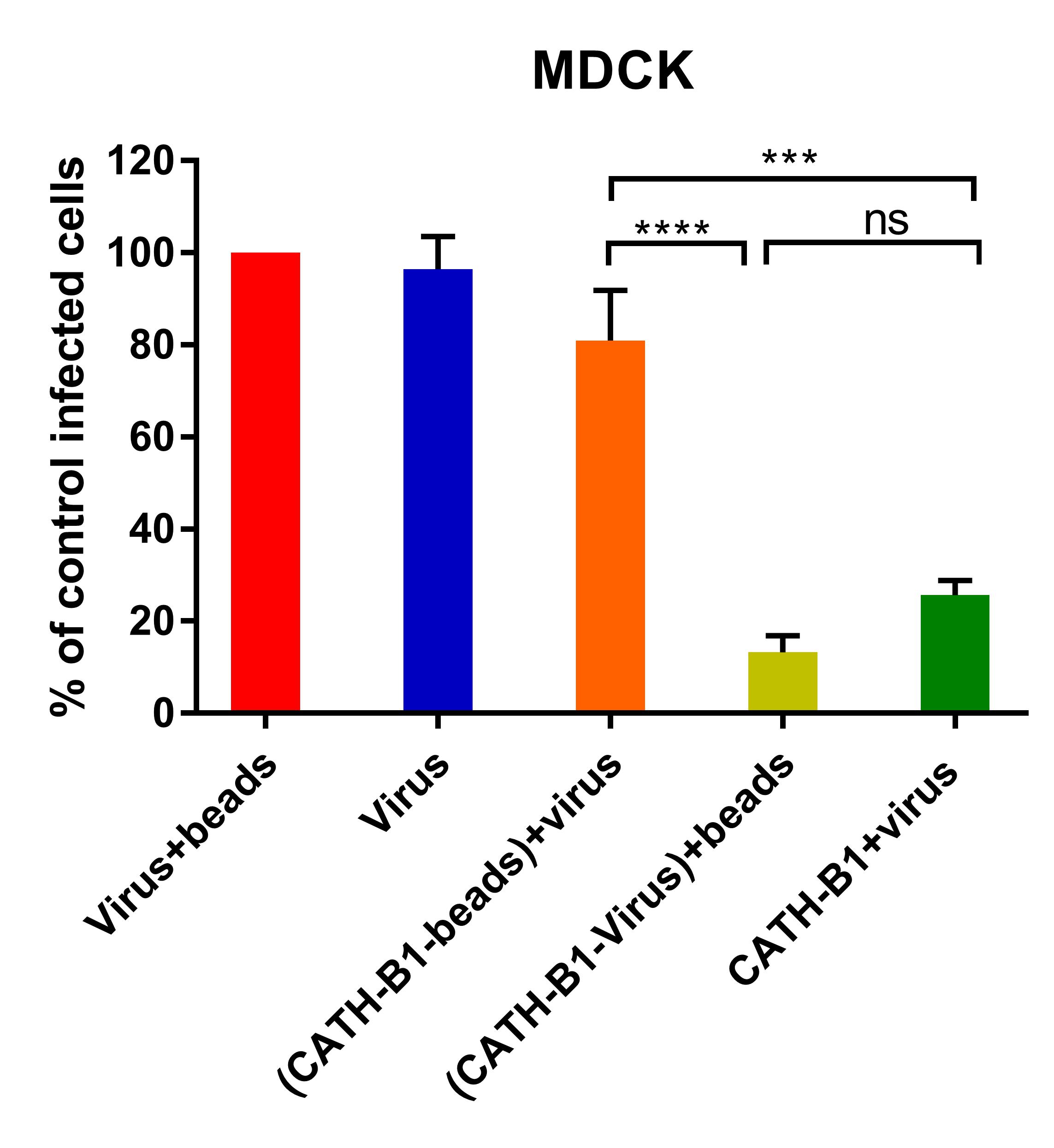

Supplement: FIGURE S3 — Binding of CATH-B1 to PR8 virus. CATH-B1 was pre-incubated with H1N1 virus after which peptide and virus were separated using Capto beads. (Virus containing) supernatant was then used to infect MDCK cells. Viral infection was determined by immunofluorescent detection of IAV nuclear protein. Three images per well were taken and the infected cells were counted. The infection rate in the presence of cathelicidins was normalized against only virus-treated wells. Data are represented as mean ± SEM of three independent experiments of triplicate samples per experiment ∗p ≤ 0.05; ∗∗p ≤ 0.01; ∗∗∗p ≤ 0.005; ****p ≤ 0.001. [file Image_3.JPEG]
